# Protease inhibitor concentrations in the saliva of individuals experiencing oral dryness

**DOI:** 10.1186/s12903-021-02024-x

**Published:** 2021-12-20

**Authors:** Kenkichi Yamamoto, Makiko Hiraishi, Mai Haneoka, Hidetake Fujinaka, Yoshitaka Yano

**Affiliations:** 1 Personal Health Care Products Research Laboratories, Kao Corporation, 2-1-3 Bunka, Sumida-ku, Tokyo, 131-8501 Japan; 2 Analytical Science Research Laboratories, Kao Corporation, Tochigi, Japan

**Keywords:** Salivary cystatins, Peptide hydrolases, Oral dryness, Xerostomia, Humans

## Abstract

**Background:**

Oral dryness is a common symptom that may interfere with swallowing, chewing, and taste. The most common reason for oral dryness is hyposalivation. Some individuals experiencing oral dryness do not have hyposalivation, however, and the reverse is also true. Here, we focused on healthy individuals with a lower salivary flow rate and evaluated the relationship between the perception of oral dryness and salivary parameters to clarify the cause underlying the perception of oral dryness.

**Methods:**

A total of 59 participants were divided into 2 groups with a lower or higher salivary flow rate according to the median salivary flow rate. In participants with a lower salivary flow rate, we assessed salivary bacterial counts, protease activities, protein concentrations, oral parameters, and the subjective perception of oral dryness.

**Results:**

Protease activities and concentrations of protease inhibitors such as cystatin-D and cystatin-SA in the saliva of participants experiencing oral dryness were significantly higher and lower, respectively, than in those not experiencing oral dryness, even though no difference in the salivary flow rate was detected. Salivary cystatin-D and cystatin-SA concentrations correlated negatively with salivary protease activities.

**Conclusions:**

The composition of salivary protease inhibitors and increased protease activities affect the subjective perception of oral dryness.

## Introduction

Saliva is important for oral health. Major saliva-producing glands include the parotid, submandibular, and sublingual glands, but minor glands located beneath the oral mucosa also contribute to salivary levels. Oral disease and discomfort may result from oral dryness. The subjective perception of oral dryness, or xerostomia, is not always associated with objectively lower salivary secretion levels [1]. The most common reasons for xerostomia are hyposalivation due to oral or systemic disease [2], head and neck radiotherapy [3], and certain medication [4]. Hyposalivation leads to an increased risk of dental caries or periodontitis, oral mucosa or tongue pain, and bacterial and fungal infections [5]. Several studies [6–8] have demonstrated a correlation between the whole salivary secretion rate and the perception of oral dryness, whereas others have not [9, 10]. In addition to hyposalivation, oral dryness can be caused by dehydration and mouth breathing [11]. The experience of oral dryness may also relate to the contribution of minor salivary glands present throughout the oral cavity, since minor labial salivary gland secretion rates were reduced among individuals with subjective oral dryness [12, 13]. Accumulation of plaque and an increase in the number of bacteria in the saliva could be expected when the salivary secretion rate is low. Lactobacilli, Candida and Streptococcus mitis were increased in the saliva of the individuals with xerostomia [14, 15].

Saliva contains an abundance of proteins, including mucin, histatin, cystatin, statherin, amylase, and proline-rich protein. These proteins are essential for maintaining tooth and mucosal integrity, and are also involved in digestion, lubrication, buffering, and antibacterial activity [16]. Several studies have evaluated changes in salivary protein levels in individuals experiencing oral dryness. Pramanik et al*.* [17] reported that mucins on the anterior tongue were reduced in patients with dry mouth. Mizuhashi et al. [18] reported reduced rates of the antimicrobial proteins lactoferrin and chromogranin A in those experiencing oral dryness. These studies, however, focused on individuals with hyposalivation experiencing oral dryness, and it is unclear whether changes in the salivary protein levels are also observed in individuals without hyposalivation but still experiencing oral dryness.

The present study, therefore, evaluated whether salivary proteins influence the perception of oral dryness in systemically healthy subjects. The subjective perception of oral dryness was examined in relation to clinical, oral, bacterial, and salivary parameters.

## Methods and materials

### Study participants

A total of 59 healthy male Japanese volunteers aged 31 to 57 years (mean 45.7 ± 6.2) participated in the study. Participants were recruited as volunteers from among employees of the Kao Corporation in Japan. All participants responded to a questionnaire regarding medication intake and general medical data. Only healthy individuals without systemic disorders or metabolic diseases were included in the study. As described below in detail, resting whole saliva was collected under the same conditions for each individual. We then divided the subjects into 2 groups: those with a lower salivary flow rate (n = 30) and those with a higher salivary flow rate (n = 29) on the basis of the overall median salivary flow rate. Participants with a lower salivary flow rate were used as study subjects. Women were not included because salivary flow rates can be influenced by the menstrual cycle [19]. All participants provided written informed consent prior to their participation in the study. The Ethics Committee of the Kao Corporation approved the study (approval number:12–10), which followed the tenets of the Declaration of Helsinki.

### Clinical and oral discomfort assessment

All experiments were performed between 8:30 AM and 11:30 AM. From the test day to the end of the test session, participants were asked to avoid activities that might influence salivary flow (e.g., eating, drinking, gargling, toothbrushing, and smoking). Participants were also asked not to consume alcohol for at least 12 h before starting the experiments. Clinical parameters of both dental and gingival health were assessed by routine methods. Decaying, missing, and filled teeth were recorded. In addition, the gingival index, bleeding on probing, probing depth, and oral hygiene index were determined as described previously [20–23]. The Winkel tongue coating index was used to score the degree of tongue coating [24]. Volatile sulfur compounds were assessed using a portable sulfide monitor (MS-Halimeter E, Interscan Corporation, Simi Valley, CA) [25]. To assess oral dryness perception, we used the following oral dryness question (with a YES/NO response option) based on the study by Farsi [26]: “Does your mouth feel dry?”.

### Collection of saliva samples

Subjects rinsed their mouths thoroughly with 6 mL of water before saliva collection. The oral rinsing water was collected to measure turbidity at the optical density of 660 nm (OD 660 nm) using a Sunrise™ microplate reader (Tecan Trading AG, Männedorf, Switzerland). Mucosal detachments were measured by centrifuging the oral rinsing solution (15,000 rpm for 15 min at 4 °C) and then measuring the wet weight of the pellet after decanting the supernatant. Resting whole saliva was collected with subjects in the seated position; subjects were asked to expectorate directly into a sterile container for 10 min. The volume of saliva (in grams) collected over the 10-min period was used to calculate the salivary flow rate (g/min). The samples were analyzed immediately for bacterial counts as described below, followed by clearance by centrifugation (3000 rpm for 15 min at 4 °C) and storage at − 80 °C until assayed.

### Biochemical and microbial analysis of saliva

The protein concentration of the resting saliva was determined using a Coomassie reagent-staining assay, using BSA as a standard. 5 μL of 5-times diluted saliva supernatant was pipetted into a clear flat-bottom 96 well plate and 250 μL of Advanced Protein Assay Reagent (Cytoskeleton Inc., Denver, CO) was added and mixed. The absorbances at 595 and 450 nm were measured. The data were processed using the ratio of the absorbance at 595 nm to that at 450 nm for all samples. Enzymatic activity of saliva, such as protease activity, was assayed using the synthetic substrate Boc-Phe-Ser-Arg-MCA (t-butyloxycarbonyl-L-phenylalanyl-L-seryl-L-arginine4-methyl-7-coumarylamide (Peptide Institute Incorporated, Osaka, Japan); final conc. 100 μM in dimethyl sulfoxide) [27, 28] in sodium phosphate buffer (pH 7.0). 100 μL of saliva supernatant was incubated at 37 °C for 10 min with 100 μL sodium phosphate buffer containing the synthetic substrate. After incubation, 7-amino-4-methylcoumarin (AMC) was measured at 460 nm (excitation at 380 nm), using AMC (Peptide Institute Incorporated, Osaka, Japan) as a standard. We defined 1 unit of enzyme activity as the amount of enzyme that produced 1 μM of AMC/mL. Salivary bacterial counts were measured using a bacteria counter (Panasonic, Osaka, Japan), using the dielectrophoresis and impedance measurement method [29]. The assays were all performed according to the manufacturers’ instructions.

### Identification of saliva proteins by mass spectrometry

For ultrafiltration, saliva samples were diluted with 100 mM Tris/HCl buffer (pH 8.0), transferred to a 3-kDa-cutoff Millipore ultrafiltration device (Merck KGaA, Darmstadt, Germany), and centrifuged at 15,000 rpm at 4 °C for 30 min. 100 mM Tris/HCl buffer (pH 8.0) was transferred to the 3-kDa-cutoff Millipore ultrafiltration device 3 times to wash the retentate. The protein concentration of concentrated saliva was determined using the Advanced Protein Assay. Saliva samples were diluted with 100 mM Tris/HCl buffer to obtain equal amounts of protein. Briefly, 90 μL aliquots were reduced by mixing with 100 mM dithiothreitol at 57 °C and incubated for 45 min. The mixture was then alkylated with 0.6 mM iodoacetamide in the dark and at room temperature for 30 min. The samples were then digested with sequencing grade-modified trypsin (1:20 [w/w] enzyme to substrate ratio; Promega, Madison, WI) at 37 °C for 19 h. Finally, formic acid (final concentration 0.3%) was added to terminate the digest.

Mass spectrometric identification of proteins was performed by a previously described method with modification [30]. Briefly, peptide analysis was performed using liquid chromatography-mass spectrometry (LC–MS; LC: Ultimate 3000 RSLCnano System, Dionex, Sunnyvale, CA; MS: quadrupole time-of-flight mass spectrometer, Triple TOF® 5600 + , AB SCIEX, Framingham, MA). The tryptic digests (1 µg of total protein from each saliva sample) were loaded onto an Acclaim PepMap 100 Nano Trap C18 (75 µm × 20 mm; particle size 3 µm, nanoViper, Dionex) at a flow rate of 5 µL/min for 5 min using 0.1% trifluoroacetic acid, and separated with nanoAcquity UPLC BEH130 C18 (100 µm × 100 mm, particle size 1.7 µm, Waters, Milford, MA) at a flow rate of 400 nL/min. Mobile phases consisted of A (0.1% formic acid) and B (80% acetonitrile/20% water containing 0.1% formic acid). A linear gradient of 2% to 50% B over 120 min was used throughout the study. Instrument control and data acquisition (information-dependent acquisition) were performed with Analyst TF 1.6 software (AB SCIEX, Framingham, MA). The ESI voltage was 2300 V, the interface temperature was 150 °C, and the declustering potential was 80 V. In an information-dependent acquisition cycle, one MS scan was performed for 250 ms, and a maximum of 20 ions with an intensity greater than 150 cps and charge states of 2, 3, 4, and 5 were selected for MS/MS scans with an accumulation time of 100 ms. The scan ranges were *m/z* 350 to 1250 for MS and *m/z* 100 to 2000 for MS/MS. The raw data for each sample were processed using Protein Pilot software 4.5 (AB SCIEX, Framingham, MA) to generate the MGF file.

### Database search and protein identification

The Mascot version 2.3 database search engine (Matrix Science, Chicago, IL) was used for protein identification against the Swiss-Prot protein database (http://www.uniprot.org/). We performed database searches for the carbamidomethylation of cysteine as a fixed modification and oxidation of methionine as a variable modification. Enzyme specificity was set to trypsin, and the allowed number of missed cleavages was set to 1. MS tolerances were 20 ppm for the precursor and 20 mmu for the fragment ions. The positive protein identification criteria were confidence levels > 99%. Mascot’s automatic decoy searches were performed to estimate the false discovery rate (FDR). Briefly, each time a protein sequence from the Swiss-Prot database was tested, a reversed decoy sequence of the same length was automatically generated and tested. The FDR was calculated as FDR = FP/(FP + TP), where FP and TP are false and true positive matches, respectively. The number of peptides matches in the target database is FP + TP and number of peptides matches in the decoy database is FP. In all proteomic analyses, an FDR < 1% was used as an identity threshold. Protein contents in molar percentages were calculated by the exponentially modified protein abundance index (emPAI), as described by Ishihama et al. [30]. Salivary antileukoproteinase and cystatins concentrations were calculated by multiplying emPAI by the total protein concentration (emPAI*mg/mL).

### Statistical analysis

Difference between values, such as salivary protease activity, salivary protein concentration, and oral parameters, in the oral dryness group and the control group were statistically analyzed using the Mann–Whitney U test. The correlations between salivary protease activities and salivary cystatins were examined using the Spearman correlation coefficient. A *p*-value less than 0.05 was considered statistically significant. BellCurve for Excel (Social Survey Research Information, Tokyo, Japan) was used for the statistical analyses.

## Results

### Resting whole salivary flow secretion

**Fig. 1:**
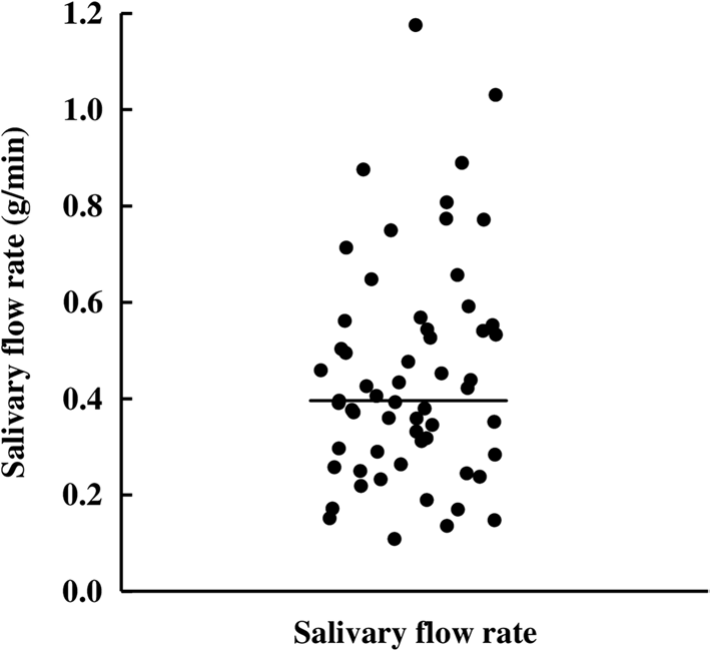
Resting whole salivary flow rates of 59 subjects. Bar represents the median

Resting whole salivary flow rates of 59 subjects are shown in dot plots, with a median of 0.40 g/min (Fig. 1). We then divided the subjects into 2 groups: those with a lower salivary flow rate (n = 30) and those with a higher salivary flow rate (n = 29) on the basis of the overall median salivary flow rate. Subsequent assessment was conducted in subjects with a lower salivary flow rate. The mean flow rate of lower salivary flow rate group and higher salivary flow rate group were 0.28 and 0.62 g/min, respectively.

### Comparison of clinical and oral parameters in subjects with and without subjective perceptions of oral dryness

**Fig. 2:**
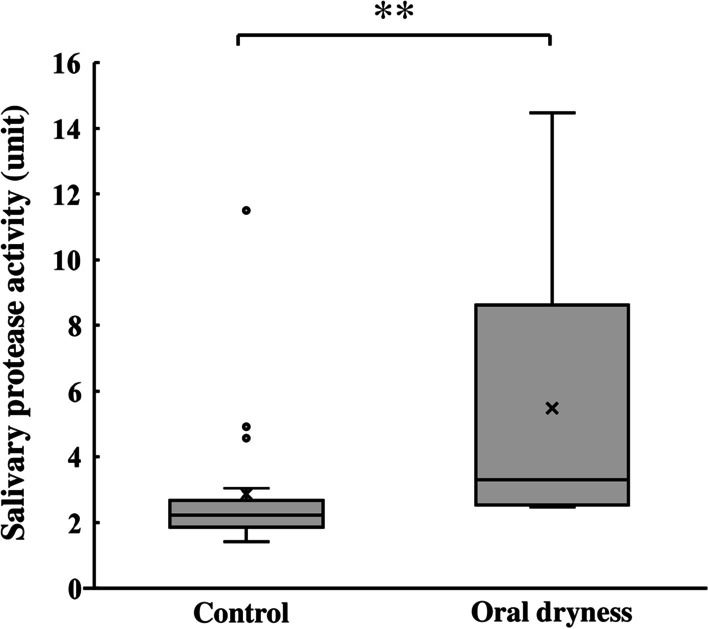
Salivary protease activity of the control group (n = 21) and oral dryness group (n = 9). Values are shown in box and whisker plots representing the median, interquartile, and range. Cross mark and circle represent the means and outliers, respectively. Mann–Whitney U test, vs control; **p < 0.01

We administered questionnaires to subjects with a lower salivary flow rate to assess the subjective perception of oral dryness. On this basis, the subjects were divided into 2 groups, 9 individuals who experienced oral dryness (oral dryness group) and 21 individuals who did not (control group). We next determined the salivary flow rate in the 2 groups (Table 1). No significant difference in the mean salivary flow rate was detected between the groups: 0.25 ± 0.08 and 0.29 ± 0.09 g/min in the oral dryness and the control groups, respectively. Because there was no difference in the salivary flow rate between the 2 groups, we next examined the clinical and oral parameters. The clinical and oral parameters are shown in Table 1. The oral dryness group had significantly higher salivary protease activities compared with the control group (Fig. 2). The median salivary protease activity in the control group and oral dryness group were 2.2 and 3.3 units, respectively. The oral dryness group also had significantly higher bleeding on probing and gingival index compared with the control group (Table 1).

**Table 1:** Comparison of oral parameters in subjects with and without the perception of oral dryness

	Control group (n = 21)	Oral dryness group (n = 9)
Age	45.8 ± 6.6	45.1 ± 5.9
Saliva flow rate (g/min)	0.29 ± 0.09	0.25 ± 0.08
Salivary protein concentration (mg/mL)	0.74 ± 0.46	0.62 ± 0.30
Salivary bacterial counts (logCFU/mL)	6.19 ± 0.48	6.30 ± 0.39
Oral rinsing solution turbidity (OD_660_)	0.25 ± 0.13	0.33 ± 0.10
Mucosal detachment (mg)	7.7 ± 2.5	9.5 ± 2.7
DMFT	12.0 ± 6.6	12.4 ± 6.5
GI	0.93 ± 0.15	1.06 ± 0.10*
BOP	0.08 ± 0.08	0.16 ± 0.10*
PD (mm)	2.50 ± 0.18	2.66 ± 0.28
OHI	1.78 ± 0.91	2.11 ± 0.77
WTCI	1.95 ± 2.29	3.44 ± 2.46
VSC (ppb)	229 ± 144	228 ± 106

Values are shown as the mean ± SD. Mann–Whitney U test, vs control; *p < 0.05

*BOP* bleeding on probing; *CFU* colony forming units; *DMFT* decayed, missing, and filled teeth; *GI* gingival index; *OD* optical density; *OHI* oral hygiene index; *PD* probing depth; *VSC* volatile sulfur compounds; *WTCI* Winkel tongue coating index

### Salivary protease inhibitors in subjects with and without subjective perceptions of oral dryness

**Fig. 3:**
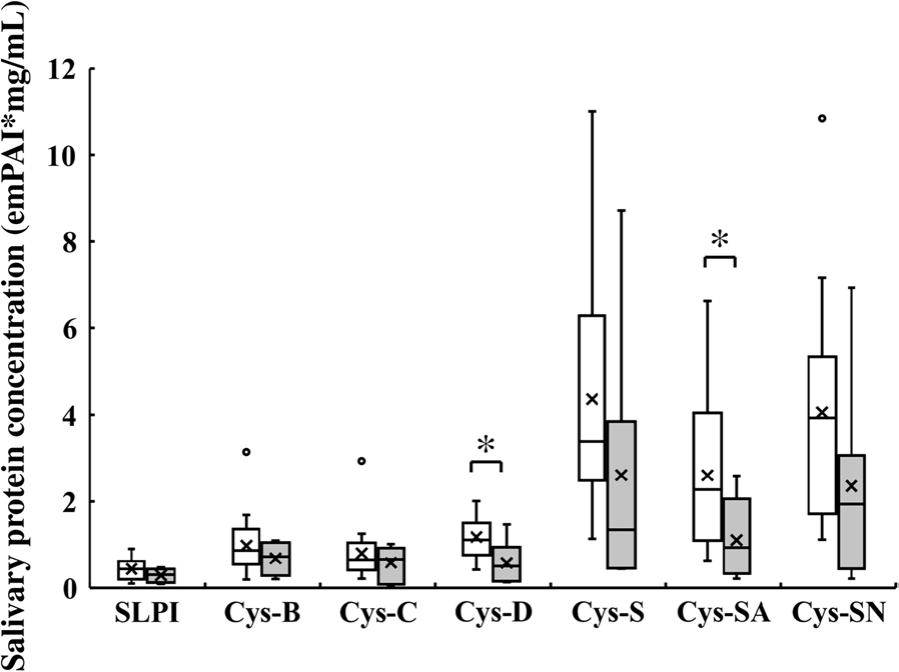
Salivary protease inhibitory proteins (antileukoproteinase [SLPI] and cystatin [Cys]) concentrations of the control group (open column) and the oral dryness group (gray column). Values are shown in box and whisker plots representing the median, interquartile, and range. Cross mark and circle represent the means and outliers, respectively. emPAI, exponentially modified protein abundance index. Mann–Whitney U test, vs control; *p < 0.05

Because the protease activities varied among the oral dryness symptom groups, we next examined the concentrations of salivary protease inhibitors by peptide analysis using LC–MS. Saliva samples from each group were assessed for the concentrations of salivary protease inhibitory proteins such as antileukoproteinase and the cysteine protease inhibitors, cystatin-B, -C, -D, -S, -SA, and -SN. Salivary cystatin-D and -SA concentrations were significantly lower in the oral dryness group than in the control group (Fig. 3). The median salivary cystatin-D and cystatin-SA concentrations in the control group were 1.11 and 2.28 emPAI*mg/mL, respectively, whereas those in the oral dryness group were 0.50 and 0.93 emPAI*mg/mL, respectively. The oral dryness group tended to have lower concentrations of salivary antileukoproteinase, and cystatin-B, -C, -S, and -SN, although the differences between the 2 groups were not significant.

### Correlations of salivary protease activity and salivary protein inhibitors

**Fig. 4:**
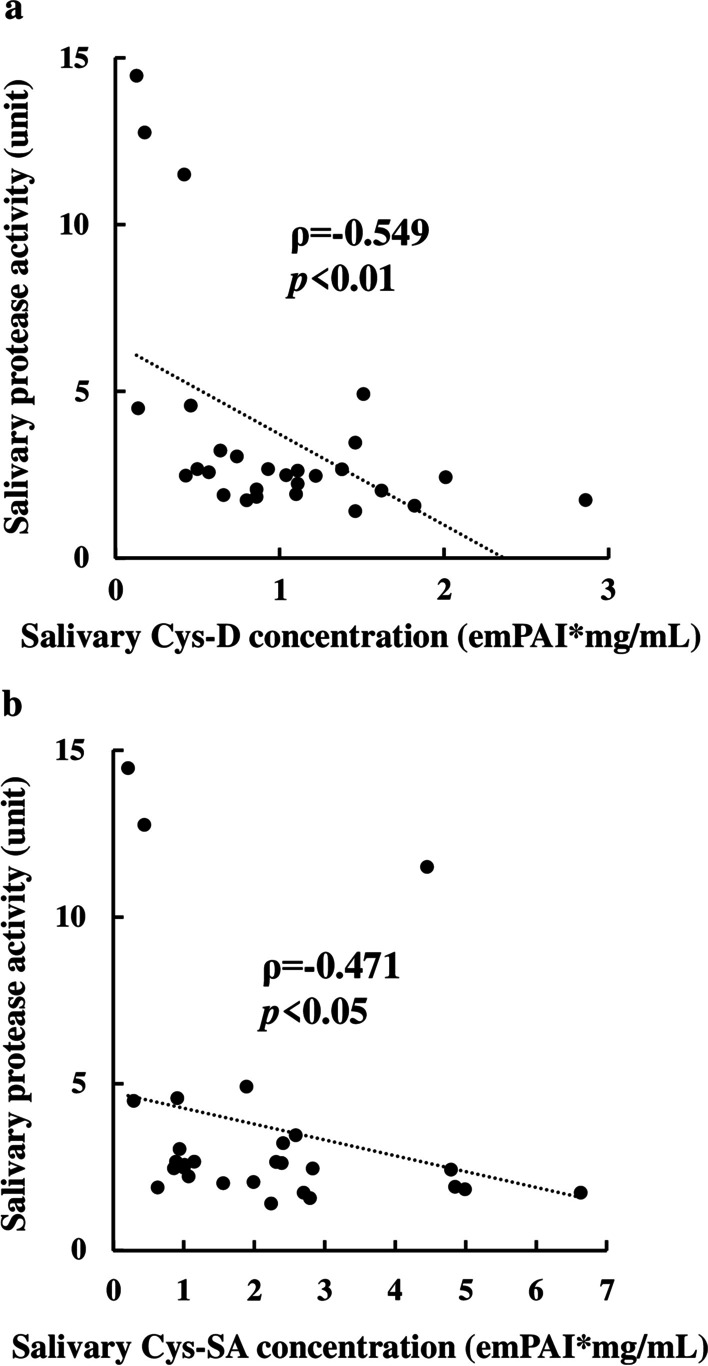
Spearman rank correlation coefficient (ρ) of salivary protease activity and salivary **a** Cys-D, or **b** Cys-SA concentrations in 30 subjects. *emPAI* exponentially modified protein abundance index

Because salivary protease activities and salivary protease inhibitors concentrations changed inversely in each group, we next examined the correlations of salivary protease activities and concentrations of salivary protein inhibitors. Salivary cystatin-D and cystatin-SA concentrations were significantly negatively correlated with salivary protease activities (Fig. 4a, b).

## Discussion

In the present study, we focused on systemically healthy individuals with relatively lower salivary flow rates to evaluate whether they experienced oral dryness. On the basis of whether they experienced oral dryness, the subjects were separated into 2 groups, those with and without the subjective perception of oral dryness. The salivary flow rate did not differ between the 2 groups. Unstimulated salivary flow rate is approximately 0.3–0.4 mL/min in normal humans, whereas the unstimulated salivary flow rate in those with hyposalivation is ≤ 0.1 mL/min [31–33]. In the present study, none of the subjects had an unstimulated salivary flow below 0.1 g/min, indicating that none of the subjects had hyposalivation. Previous studies mentioned that some individuals experience oral dryness even when the salivary secretion rate is normal [9, 10]. Therefore, the underlying cause of the perception of oral dryness was thought to be an issue other than a decrease in the salivary flow rate.

We found that salivary protease activities were higher in subjects experiencing oral dryness than those not experiencing oral dryness. We further demonstrated that the salivary cystatin-D and -SA concentrations were lower in the oral dryness subjects than in the control subjects. The concentrations of salivary cystatin-D and cystatin-SA showed significant negative correlations with salivary protease activities. These observations suggest that activation of proteases caused by the reduction of inhibitory protease expression is involved in the perception of oral dryness.

The mechanism underlying the perception of oral dryness is complicated. Analysis of clinical parameters revealed that gingival index and bleeding on probing in subjects experiencing oral dryness were significantly higher than in those not experiencing oral dryness. Cystatins have antimicrobial and protease inhibitory properties [34, 35]. Therefore, it is likely that the build-up of periodontitis-associated bacteria on the gingiva occurs, leading to gingival inflammations in the subjects with low concentrations of salivary protease inhibitors. *Porphyromonas gingivalis* is an anaerobic gram-negative bacterium frequently isolated from advanced periodontal lesions [36]. *P. gingivalis* has 2 major cysteine proteinases, Arg-specific gingipain and Lys-specific gingipain [27], which possess an extracellular matrices decomposition function, such as type I and IV collagen, fibronectin, and laminin [27, 37]. Because human salivary cystatin-SA exhibits antimicrobial effects against the *P. gingivalis* [34], such a decomposition of the extracellular matrix caused by low levels of cystatins, including cystatin-SA, in saliva appears to be associated with the perception of oral dryness. Oral hygiene products containing antimicrobial proteins have been reported to reduce gingival inflammation and mucosal irritation, and to improve the subjective symptoms of dry mouth in patients with xerostomia [38–41]. Oral mucosal conditions may lead to a perception of oral dryness, although further studies are needed.

This study has a few limitations. First, the number of participants was relatively small. While a larger sample would have been desirable, the fact that there was sufficient difference to obtain statistical significance indicated that the sample size was at least adequate for the analysis to find large differences with a limited sample size. The second, the participants were limited to the lower salivary flow rate subjects. Finally, the identification of salivary protein by MS was not validated using immunochemistry technique, such as western blot or ELISA. Within limitations, this study identified the composition of salivary protease inhibitors and increased protease activities affect the subjective perception of oral dryness. This study can serve as a baseline observation for future comprehensive studies, which could identify the role of salivary protease inhibitors in the perception of oral dryness.

## Conclusions

Low concentrations of protease inhibitors such as cystatin-D and cystatin-SA were detected by LC–MS in the saliva of healthy individuals who experienced oral dryness. The composition of salivary protease inhibitors and increased protease activities affect the subjective perception of oral dryness. Control of salivary protease activities appears to be necessary for improvement of the quality of life of individuals experiencing oral dryness.

## Data Availability

The datasets obtained and analyzed during the current study are available from the corresponding author on reasonable request.

## Abbreviations

LC–MS: Liquid chromatography–mass spectrometry
FDR: False discovery rate
emPAI: Exponentially modified protein abundance index
